# Supplementary material for: Undernutrition combined with dietary mineral oil hastens depuration of stored dioxin and polychlorinated biphenyls in ewes. 2. Tissue distribution, mass balance and body burden
Source: PLoS One. 2020 Mar 31;15(3):e0230628. doi: 10.1371/journal.pone.0230628 (PMC7108722; doi:10.1371/journal.pone.0230628)
Supplement: S1 Table — (DOCX) [file pone.0230628.s001.docx]

| **Table S1. Empty body chemical composition and adipocyte volume of ewes at slaughter (end of depuration period, day +57)^1^** | | | | |
| --- | --- | --- | --- | --- |
| Item | Treatment | | SEM | *P*-value |
|  | CTL | UFMO |  |  |
| Empty body chemical composition^2^ | | | | |
| Total weight (kg) | 47.8 | 40.9 | 2.7 | 0.10 |
| Water (kg) | 27 | 23.2 | 1.2 | 0.06 |
| Dry matter (kg) | 20.8 | 17.8 | 1.8 | 0.28 |
| Lipids (kg) | 11.8 | 9.8 | 1.6 | 0.43 |
| Proteins (kg) | 7.5 | 6.3 | 0.4 | < 0.001 |
| Ashes (kg) | 1.9 | 1.9 | 0.1 | 0.83 |
| Energy (Mcal) | 151 | 125 | 16 | 0.28 |
| Adipocyte volume (pL) | | | | |
| Mesenteric | 500 | 429 | 57 | 0.36 |
| Perirenal | 578 | 447 | 133 | 0.52 |
| Pericaudal subcutaneous | 263 | 227 | 40 | 0.55 |
| **^1^**Four ewes received a control well-fed and non-supplemented treatment (CTL), while five ewes received an underfed and mineral oil supplemented treatment (UFMO).  ^2^Empty body: total body minus gut contents and wool. | | | | |
