# Supplementary material for: Undernutrition combined with dietary mineral oil hastens depuration of stored dioxin and polychlorinated biphenyls in ewes. 2. Tissue distribution, mass balance and body burden
Source: PLoS One. 2020 Mar 31;15(3):e0230628. doi: 10.1371/journal.pone.0230628 (PMC7108722; doi:10.1371/journal.pone.0230628)
Supplement: S2 Table — (DOCX) [file pone.0230628.s002.docx]

| **Table S2. Weights of ewes body compartments at slaughter and of oral intakes, and output compartments over the depuration period^1^** | | | | | | | | | |
| --- | --- | --- | --- | --- | --- | --- | --- | --- | --- |
| Weight | Intake (kg) | Body compartments (g) | | | | | | Output compartments | |
|  |  | Adipose tissue | | | Liver | *Rect. abdo.* Muscle^3^ | Serum | Feces (kg) | Wool (g) |
|  |  | Mesenteric | Perirenal | Pericaudal sc.^2^ |  |  |  |  |  |
| Fresh matter | | | | | | | | | |
| CTL | 60 | 871 | 617 | 116 | 631 | 187 | 2,500 | 89 | 194 |
| UFMO | 39 | 603 | 457 | 70 | 567 | 147 | 2,200 | 69 | 115 |
| SEM | 3 | 97 | 180 | 20 | 40 | 17 | 100 | 18 | 14 |
| *P*-value | < 0.001 | 0.09 | 0.64 | 0.16 | 0.29 | 0.14 | 0.07 | 0.47 | < 0.01 |
| Dry matter | | | | | | | | | |
| CTL | 54 | 724 | 574 | 93 | 189 | 51 | *Not determined* | 23 | 176 |
| UFMO | 36 | 499 | 419 | 57 | 177 | 40 |  | 21 | 106 |
| SEM | 2 | 97 | 134 | 20 | 14 | 5 |  | 2 | 13 |
| *P*-value | < 0.001 | 0.15 | 0.45 | 0.26 | 0.54 | 0.16 |  | 0.50 | < 0.01 |
| Lipids | | | | | | | | | |
| CTL | 0.9 | 699 | 556 | 86 | 33 | 13 | 6.0 | 0.5 | 15 |
| UFMO | 3.3 | 483 | 406 | 54 | 38 | 10 | 4.3 | 2.7 | 12 |
| SEM | 0.1 | 93 | 169 | 20 | 5 | 1 | 0.3 | 0.1 | 2 |
| *P*-value | < 0.001 | 0.15 | 0.64 | 0.29 | 0.43 | 0.23 | < 0.01 | < 0.001 | 0.30 |
| Proteins | | | | | | | | | |
| CTL | 3.3 | 24 | 17.4 | 6 | 146.5 | 36 | *Not determined* | *Not determined* | *Not determined* |
| UFMO | 1.5 | 16 | 12.2 | 3 | 130.2 | 28 |  |  |  |
| SEM | 0.1 | 8 | 6 | 1 | 33 | 3 |  |  |  |
| *P*-value | < 0.001 | 0.44 | 0.57 | 0.03 | 0.75 | 0.17 |  |  |  |
| Ashes | | | | | | | | | |
| CTL | 3.6 | 1.2 | 0.4 | 0.3 | 10.1 | 2.1 | *Not determined* | 2.4 | *Not determined* |
| UFMO | 3.2 | 0.9 | 0.4 | 0.1 | 8.4 | 1.5 |  | 2.0 |  |
| SEM | 0.2 | 0.1 | 0.1 | 0.0 | 0.6 | 0.2 |  | 0.2 |  |
| *P*-value | 0.17 | 0.17 | 0.67 | 0.13 | 0.10 | 0.03 |  | 0.17 |  |
| **^1^**Four ewes received a control well-fed and non-supplemented treatment (CTL), while five ewes received an underfed and mineral oil supplemented treatment (UFMO).  ^2^Pericaudal sc.: pericaudal subcutaneous. ^3^*Rect. abdo*. muscle: *Rectus abdominis* muscle. | | | | | | | | | |
