# Supplementary material for: Undernutrition combined with dietary mineral oil hastens depuration of stored dioxin and polychlorinated biphenyls in ewes. 2. Tissue distribution, mass balance and body burden
Source: PLoS One. 2020 Mar 31;15(3):e0230628. doi: 10.1371/journal.pone.0230628 (PMC7108722; doi:10.1371/journal.pone.0230628)
Supplement: S3 Table — (DOCX) [file pone.0230628.s003.docx]

| **Table S3. Dioxin (TCDD) and polychlorinated biphenyls (PCBs) concentrations in body tissues of ewes at slaughter and in output compartments over the depuration period^1^** | | | | | | | | | | | | | | | | | | | |
| --- | --- | --- | --- | --- | --- | --- | --- | --- | --- | --- | --- | --- | --- | --- | --- | --- | --- | --- | --- |
| Pollutant  concentrations  (/g lipids) | Treatment | Body compartments | | | | | | | | | | | Output compartments | | | SEM | *P*-value | | |
|  |  | Empty body^2^ | Adipose tissues | | | | | | Liver | *Rect. abdo.* muscle^4^ | | Blood serum | Faeces | | Wool |  | Trt. | Comp. | Trt. × Comp. |
|  |  |  | Mesenteric | | Perirenal | | Pericaudal sc.^3^ | |  |  |  |  |  |  |  |  |  |  |  |
| TCDD (pg) | CTL | 20.9^bc^ | 20.9^bc^ |  | 22.4^b^ | * | 22.1^bc^ | * | 50.7^a^ | 19.5^bc^ | † | 7.4^d^ | 15.7^c^ | * | 25.4^b^ | 3.0 | 0.44 | <0.001 | <0.01 |
|  | UFMO | 25.1^c^ | 25.0^c^ |  | 31.9^b^ |  | 30.5^bc^ |  | 53.1^a^ | 26.7^c^ |  | 8.3^d^ | 6.2^d^ |  | 29.0^bc^ |  |  |  |  |
| PCB 126 (pg) | CTL | 19.3^d^ | 19.7^d^ |  | 22.7^d^ |  | 20.2^d^ |  | 144.6^a^ | 18.4^d^ | † | 13.8^d^ | 35.0^c^ | ** | 55.7^b^ | 3.4 | 0.85 | <0.001 | <0.001 |
|  | UFMO | 26.1^cd^ | 29.7^c^ |  | 31.4^c^ |  | 30.7^c^ |  | 132.4^a^ | 23.5^cd^ |  | 18.7^de^ | 9.5^e^ |  | 55.7^b^ |  |  |  |  |
| PCB 153 (ng) | CTL | 22.3^b^ | 21.0^b^ | † | 29.5^b^ | * | 22.5^b^ | * | 27.9^b^ | 24.2^b^ |  | 6.5^c^ | 20.5^b^ | ** | 40.9^a^ | 1.5 | 0.38 | <0.001 | <0.01 |
|  | UFMO | 25.1^c^ | 28.6^bc^ |  | 41.0^a^ |  | 31.3^abc^ |  | 28.5^bc^ | 27.2^c^ |  | 8.0^d^ | 7.3^d^ |  | 35.9^ab^ |  |  |  |  |
| ^1^Four ewes received a control well-fed and non-supplemented treatment (CTL), while five ewes received an underfed and mineral oil supplemented treatment (UFMO).  ^2^Empty body: total body minus gut contents and wool.  ^3^Pericaudal sc.: Pericaudal subcutaneaous.  ^4^*Rect. abdo*. muscle: *Rectus abdominis* muscle.  ^a-e^Means within a row with different letters differ at *P* ≤ 0.05.  *, **, †, Means within compartment (in column) and pollutant differ between treatment at *P* ≤ 0.05 (*), *P* ≤ 0.01 (**) or tended to differ at *P* ≤ 0.10 (†). | | | | | | | | | | | | | | | | | | | |
