# Supplementary material for: Undernutrition combined with dietary mineral oil hastens depuration of stored dioxin and polychlorinated biphenyls in ewes. 2. Tissue distribution, mass balance and body burden
Source: PLoS One. 2020 Mar 31;15(3):e0230628. doi: 10.1371/journal.pone.0230628 (PMC7108722; doi:10.1371/journal.pone.0230628)
Supplement: S2 File — (DOCX) [file pone.0230628.s005.docx]

**S2 File. Codes for statistical analyses.**

Data were analysed by ANOVA using the MIXED procedure of SAS 9.3.

- Code for total and empty body weights and composition, body condition score, adipocyte measurements, POPs amounts intake or excreted through faeces or wool, and POPs burdens of empty body and individual tissues analysed separately for day 0 and day +57 (example of total body weight: BW):

proc mixed ratio covtest ;

class ewe treatment;

model BW = treatment / DDFM=KenwardRoger outp=residy residual ;

random ewe ;

lsmeans treatment / pdiff ;

run;

- Code for POPs concentrations for all body tissues measured at slaughter, and faeces and wool concentrations along the depuration period (example of TCDD concentration: [TCDD]):

proc mixed ratio covtest ;

class ewe treatment compartment;

model [TCDD] = treatment compartment treatment*compartment / DDFM=KenwardRoger outp=residy residual ;

repeated compartment / type= ar(1) sub = ewe ;

random ewe ;

lsmeans treatment / pdiff ;

lsmeans compartment / pdiff adjust = Tukey;

lsmeans treatment*compartment / pdiff adjust = Tukey slice=compartment;

run;
