# Supplementary material for: Undernutrition combined with dietary mineral oil hastens depuration of stored dioxin and polychlorinated biphenyls in ewes. 2. Tissue distribution, mass balance and body burden
Source: PLoS One. 2020 Mar 31;15(3):e0230628. doi: 10.1371/journal.pone.0230628 (PMC7108722; doi:10.1371/journal.pone.0230628)
Supplement: Attachment — Submitted filename: Response to Reviewers.docx [file pone.0230628.s006.docx]

**Subject: Manuscript PONE-D-19-30218: “Undernutrition combined with dietary mineral oil hastens depuration of stored dioxin and polychlorinated biphenyls in ewes. 2. Tissue distribution, mass balance and body burden“**

Dear Editor,

We appreciate all of the constructive comments and thoughtful suggestions provided by the referee. All suggestions arising from the peer-review have been carefully considered during revision of the original manuscript. In all cases, the suggested changes have been carefully considered and implemented in full.

The associate changes are highlighted in red thorough the manuscript labeled 'Revised Manuscript with Track Changes’. A response to the comment from the referee is outlined in the accompanying response indicated by sentences starting with AU:. The line number reported in the response are the one of the 'Revised Manuscript with Track Changes’.

We hope that this revision would allow the manuscript to be considered acceptable for publication.

With our best regards,

S. Lerch,

On behalf of all co-authors

**Reviewers Comments to the Author**

Reviewer #1: In the submitted manuscript the authors investigated the efficacy of a depuration protocol, including undernutrition and mineral oil administration, in ewes dietary exposed to TCDD, PCB126 and PCB153. Chemical analyses were performed in different body tissues, as well as in feces and wool.
The study is well designed and performed with appropriate techniques. However, there are some minor concerns that should be considered before publishing:
1. Why did the authors select the investigated congeners? An explanation for their choice, which includes 2 DL-compounds and 1 NDL, would be noteworthy.

AU: We agree that no justification about the choices of these three specific molecules among the dioxins and PCBs families was included in the introduction section of the initial submission. A sentence was added at the end of the introduction section in order to justify this choice. See lines lines 62-65.

2. The paper is quite well written; however, to be suitable for publication, the manuscript should undergo English editing by a native English speaker.

AU: The R1 version of the manuscript was subjected to an additional English editing step provide by the Scribendi services (https://www.scribendi.com/). See the concomitant changes along the main text highlighted in green.

3. Lines 221-224: please, rephrase since the sentence is too long and hard to read.

AU: The sentence was split in two and rewrite accordingly. See lines 234-238.

4. Table 1: please check the values of the “changes during depuration period”, since in some cases they are not correct.

AU: Thank you for notice this. The lsmeans and *P* value of the model for empty body lipids mass at day +57 were not correct initially. Table 1 was corrected accordingly. Moreover, in the case of pericaudal adipose cell diameter data for the CTL treatment: difference between lsmeans reported for day 0 and 57 is of -3 µm, but a value of -4 µm is reported for the delta model. This is simply due to the rounding of the data (exact results are -3.75 µm). See the concomitant changes throughout the Table 1.

5. Lines 284-288: the higher accumulation of DL-compounds (and not NDL PCBs) in sheep liver compared to other species, including other ruminants, is well known. However, the reason for such phenomenon is likely not related to higher hepatic CYP1A levels, since in ovine they are lower than in bovine. In my opinion the authors should revise this part of the discussion, providing other references.

AU: We agree and further remove this sentence. Now, we only state the consistency of the results of our study when compared to previous one reporting dioxins and PCBs liver concentrations in bovine and ovine. We did not provide any more putative mechanistic explanations for liver sequestration, as the results of our study did not allow us to support any specific hypotheses. See lines 299-302.

6. Lines 290-291: In my opinion the sentence is not clear. Please, rephrase.

AU: The sentence has been rewritten accordingly the comment. See lines 304-306.

7. Lines 388-395: the authors drew a general conclusion that refers broadly to all POPs and all livestock species. Although their data are interesting and robust, in my opinion they should be cautious in extending their results to other species and POPs, due to the well-known kinetics differences. Accordingly, in the abstract (lines 29 and 43), “lipophilic food contaminants” are too generic.

AU: We agree and further adjusted the concluding statements. See lines 405-415. Moreover, terminology was reviewed throughout the text, retaining only “persistent organic pollutants” and no more “lipophilic contaminants”. See concomitant changes thorough the abstract and the text.
